# A fusion protein containing a lepidopteran-specific toxin from the South Indian red scorpion (*Mesobuthus tamulus*) and snowdrop lectin shows oral toxicity to target insects

**DOI:** 10.1186/1472-6750-6-18

**Published:** 2006-03-16

**Authors:** Nghia Pham Trung, Elaine Fitches, John A Gatehouse

**Affiliations:** 1 School of Biological and Biomedical Sciences, University of Durham, Science Laboratories, South Road, Durham DH1 3LE, UK; 2 Central Science Laboratory, Department for Environment, Food and Rural Affairs, Sand Hutton, York YO41 1LZ, UK; 3 Department of Biotechnology, Cuu Long Delta Rice Research Institute, O Mon, Can Tho, Viet Nam

## Abstract

**Background:**

Despite evidence suggesting a role in plant defence, the use of plant lectins in crop protection has been hindered by their low and species-specific insecticidal activity. Snowdrop lectin (*Galanthus nivalis *agglutinin; GNA) is transported to the haemolymph of insects after oral ingestion, and can be used as a basis for novel insecticides. Recombinant proteins containing GNA expressed as a fusion with a peptide or protein, normally only toxic when injected into the insect haemolymph, have the potential to show oral toxicity as a result of GNA-mediated uptake.

**Results:**

A gene encoding a toxin, ButaIT, from the red scorpion (*Mesobuthus tamulus*) was synthesised and assembled into expression constructs. One construct contained ButaIT alone, whereas the other contained ButaIT fused N-terminally to a GNA polypeptide (ButaIT/GNA). Both recombinant proteins were produced using the yeast *Pichia pastoris *as an expression host, and purified. Recombinant ButaIT and ButaIT/GNA were acutely toxic when injected into larvae of tomato moth (*Lacanobia oleracea*), causing slow paralysis, leading to mortality or decreased growth. ButaIT/GNA was chronically toxic when fed to *L. oleracea *larvae, causing decreased survival and weight gain under conditions where GNA alone was effectively non-toxic. Intact ButaIT/GNA was detected in larval haemolymph from insects fed the fusion protein orally, demonstrating transport of the linked polypeptide across the gut. Proteolysis of the fusion protein was also observed. ButaIT/GNA was significantly more toxic that GNA alone when fed to the homopteran *Nilaparvata lugens *(rice brown planthopper) in liquid artificial diet.

**Conclusion:**

The ButaIT/GNA recombinant fusion protein is toxic to lepidopteran larvae both when injected and when fed orally, showing the utility of GNA as a carrier to transport potentially toxic peptides and proteins across the insect gut. Although ButaIT has been claimed to be lepidopteran-specific, the fusion protein has more wide-ranging insecticidal activity. Fusion proteins based on plant lectins have potential applications in crop protection, both as exogenously applied treatments and as endogenous products in transgenic plants.

## Background

Scorpion venoms are a rich source of polypeptides with diverse biological activities, including many neurotoxins that exert their action via target-specific modulation of ion channel function (reviewed in [1,2]). Among the well-characterised peptide toxins are those derived from the venom of scorpions belonging to the family Buthidae. These toxins are classified into two groups, based on their molecular size and activity. The first group contains short toxins (30–40 amino acid residues), with 3–4 disulphide bridges, which principally affect voltage-dependent potassium channels and conductance calcium-activated potassium channels [3,4]. The second group contains longer toxins (60–70 amino acid residues), cross-linked by 4 disulphide bridges, which mainly effect voltage-dependent sodium channels of excitable cells [5,6]. Despite the similarity of invertebrate and vertebrate ion channels, many scorpion neurotoxins exhibit specificity towards particular species or groups of species. This specificity of action gives scorpion toxins, and other proteins with similar properties, significant potential in the development of safer alternatives to broad spectrum insecticides. Recombinant baculovirus expressing scorpion toxins have been proposed as insect control agents, and have given promising results in laboratory, glasshouse and field, although this has not led to wide-scale adoption of the technology (reviewed in [7]).

The isolation and characterization of a novel short, lepidopteran-selective toxin (ButaIT; also referred to as BtChl2; Swiss-Prot [P81761]) from the venom of the South Indian red scorpion (*Mesobuthus tamulus*) was reported by Wudayagiri et al. [8]. Injection of *Heliothis virescens *with ButaITA (purified to homogeneity by hplc; fraction CM-IV-6A) was shown to cause larval mortality through progressive, irreversible, flaccid paralysis at a dose of 1 μg/100 mg, but was non-toxic to blowfly larvae at the same dose, or mice at a dose of 3 μg/g body weight. The mature form of ButaIT is a single polypeptide of 37 amino acid residues, cross-linked by four disulfide bridges, that exhibits high sequence similarity to other short toxins. The sequence of a cDNA encoding a precursor form of ButaIT (referred to as BtChl2) has been lodged in the databases (Genbank [AF481881]); the precursor is a polypeptide of 62 amino acids, which is modified by removal of a 24-residue N-terminal signal peptide, and a single residue from the C-terminus, to give the mature protein.

Snowdrop lectin (GNA; *Galanthus nivalis *agglutinin) has been shown to bind to the gut epithelium when fed to insects, and can be detected in the circulatory system [9]. The use of GNA as a carrier to transport fused proteins to the haemolymph of target species after oral ingestion has been demonstrated, and can result in insecticidal effects not shown by ingestion of either the lectin or the fused protein separately. When a fusion protein containing GNA and a C-terminally fused insect neuropeptide (*Manduca sexta *allatostatin) was fed to lepidopteran larvae, reductions in survival, and in growth and feeding, were observed [10] In addition, fusion protein could be detected in the larval haemolymph by immunoassay. Similarly, GNA has been used to deliver an N-terminally fused insect-specific neurotoxin, Sfl1, derived from the venom glands of the spider *Segestria florentina*, to the blood of target species, causing insecticidal effects [11].

This paper reports on the production, purification and biological activities of a recombinant fusion protein, ButaIT/GNA, containing the ButaIT polypeptide fused to the N-terminus of snowdrop lectin, as a means of delivering ButaIT to insects via oral ingestion. The ButaIT/GNA fusion protein was toxic towards lepidopteran larvae both when injected into the haemolymph, and when fed in artifical diet. Direct evidence for the delivery of intact ButaIT/GNA to the haemolymph of orally-fed insects is presented. In contrast to the earlier results showing that ButaIT was specifically toxic to lepidopteran insects, the ButaIT/GNA fusion protein was found to be toxic towards insects of different orders.

## Results

### Construction of a synthetic gene encoding the red scorpion neurotoxin ButaIT and assembly of expression constructs

A synthetic gene containing the entire coding sequence for the mature ButaIT polypeptide (as given in Genbank [AF481881]) was assembled from overlapping oligonucleotides. Each of the two complementary strands of the gene contained four 30-mers and one 15-mer, arranged so that overlaps of 15 bases between complementary portions of the oligos were present. The oligonucleotides were phosphorylated prior to annealing, and the desired product was amplified by PCR after annealing and ligating, using the 15-mer oligonucleotides at the 5' end of each strand as primers. A DNA fragment corresponding to the correct size for the complete product was obtained, and was excised from gel, cloned in an intermediate vector, and verified by DNA sequencing. The resulting fragment corresponded to the desired coding sequence, with additional restriction sites introduced at the ends to allow for subsequent cloning.

**Figure 1:**
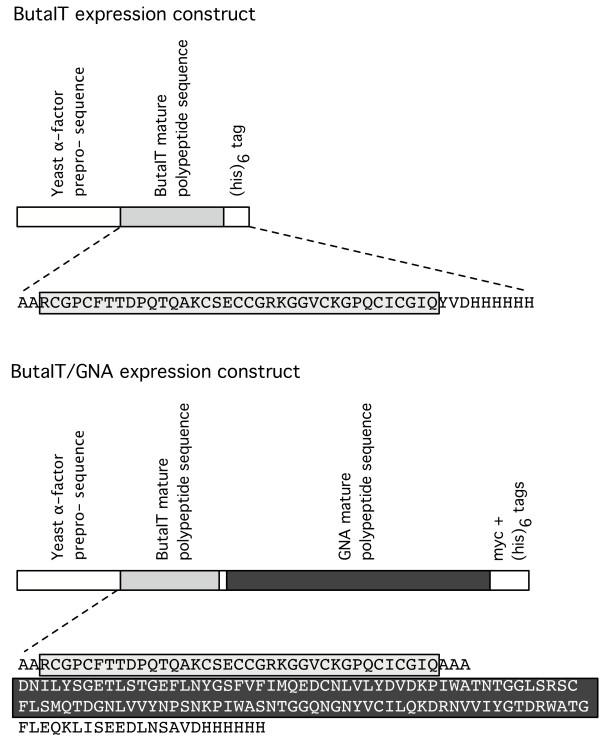
**Assembly of the expression constructs for ButaIT and ButaIT/GNA**. The predicted sequences of the mature proteins produced by the *P. pastoris *expression system are shown.

The ButaIT coding sequence was assembled into two constructs for expression of proteins in the yeast *Pichia pastoris *using the shuttle expression vector pGAPZαB. The first construct contained ButaIT alone, and the second encoded a fusion protein in which ButaIT is fused to the N-terminus of residues 1 – 105 of mature GNA via a 3 amino acid linker peptide (Fig. 1). Both constructs were arranged so that the recombinant proteins were produced as precursors containing the yeast α-factor prepro-sequence at the N-terminus; this sequence directs the product into the secretory pathway in the expression host, and is removed during translation and subsequent transport. The final protein product for ButaIT is predicted to contain a 9 amino acid C-terminal extension, containing the (his)_6 _tag, and is predicted to have 2 extra alanine residues at the N-terminus after removal of the prepro- sequence, The predicted protein product for ButaIT/GNA contains a C-terminal extension of 23 amino acids, including a *myc *epitope tag and the (his)_6 _tag (Fig. 1). Constructs were assembled by restriction/ligation and cloning in *E. coli*, and were verified by DNA sequencing.

### Expression and purification of recombinant ButaIT and ButaIT/GNA

DNA from verified clones of the expression construct plasmids was linearised and transformed into competent cells of a protease-deficient *Pichia pastoris *strain. Since the expression vector contains a constitutive yeast promoter, clones selected as positive for transformation by antibiotic resistance and colony PCR were tested for expression of recombinant proteins in small-scale cultures. Culture supernatants were assayed for the presence of recombinant proteins by immuno-dot blot and Western blotting, using antibodies to the C-terminal tags, or in the case of the ButaIT/GNA fusion, to GNA. Clones expressing recombinant proteins at higher levels (relative to other clones) were selected for further study.

**Figure 2:**
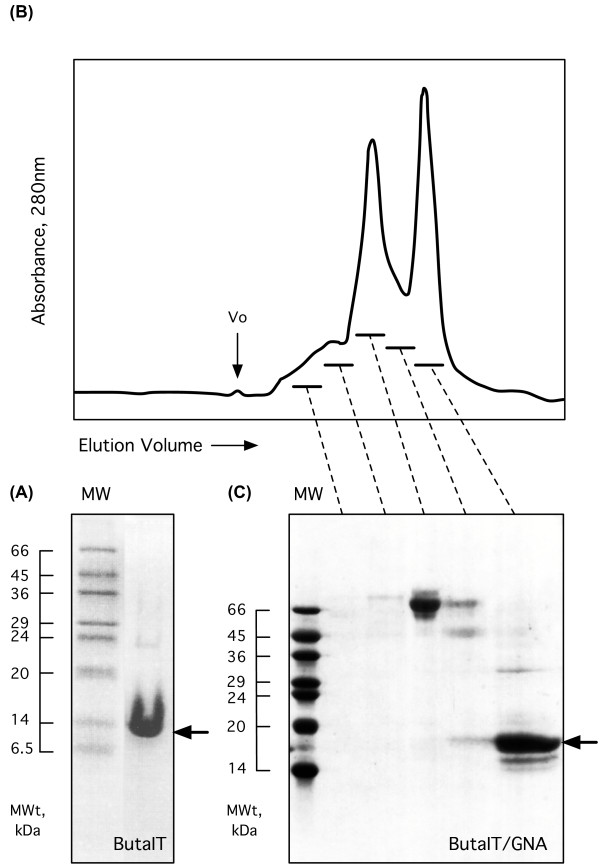
**Production of recombinant proteins**. (A). Analysis of partially purified ButaIT by SDS-PAGE (Tris-Tricine buffer system); gel stained with Coomassie blue. Arrow indicates ButaIT polypeptide, based on reaction with anti-(his)_6 _antibodies on Western blot. (B), (C). Purification of ButaIT/GNA by gel filtration. Following partial purification by phenyl-Sepharose chromatography recombinant proteins were loaded onto a Sephacryl S200 column equilibrated in PBS buffer. (B) absorbance profile and (C) corresponding fractions run on SDS-PAGE (15 % acrylamide gel, stained with Coomassie blue). The arrow depicts purified ButaIT/GNA.

For production of recombinant proteins, selected yeast clones expressing ButaIT and ButaIT/GNA were grown in a bench-top fermenter. ButaIT and ButaIT/GNA were partially purified from culture supernatant by hydrophobic interaction chromatography on phenyl-Sepharose. Recombinant ButaIT was produced in relatively low yield (<1 mg/l culture). The partially purified material contained a polypeptide of estimated mol. wt. approx. 10,000 as the major component (fig. 2A); when a gel separation was analysed by Western blotting, and probing with with anti-(his)_6 _antibodies, the band gave a strong and specific reaction, showing that this band represents recombinant ButaIT. The discrepancy in size between predicted and estimated mol. wts. for this polypeptide is consistent with previous results in which small toxin polypeptides with high cysteine content show anomalous migration on SDS-PAGE.

ButaIT/GNA was produced at a yield of 25–35 mg/l culture medium, and was the major protein component of the material eluted from the hydrophobic interaction column by a water wash. Further purification of ButaIT/GNA by gel filtration was carried out to remove contaminating yeast proteins (fig. 2B). Analysis of purified ButaIT/GNA by SDS-PAGE (Fig. 2C) shows one major component of approx. 18.5 kDa, which is close to the predicted molecular weight of 19.4 kDa. A minor component (weakly stained band) of approx. 17 kDa is also present. Both bands were immunoreactive towards anti-GNA antibodies (data not presented), but only the major component reacted with anti-(his)_6 _antibodies. The indicated size of these polypeptides, and their immunoreactivity, suggest that the majority of ButaIT/GNA is full-length protein with the C-terminal extension intact, with a small proportion of ButaIT/GNA from which the C-terminal his tag has been removed. Recombinant ButaIT/GNA agglutinated rabbit erythrocytes at a comparable concentration to native GNA (50 μg/ml for ButaIT/GNA, vs. approx. 10 μg/ml for GNA), showing that the lectin part of the fusion protein is functional.

While the ButaIT/GNA could be produced as intact fusion protein, prolonged exposure to proteases, such as those in yeast culture supernatant, resulted in varying amounts (normally <50%, as estimated by band staining intensity) of proteolytic cleavage. Proteolysis occurred predominantly at or near the linker region between ButaIT and GNA, and generated a polypeptide, mol. wt. approx. 12,000, which reacted with anti-GNA and anti-(his)_6 _antibodies. When band intensities were compared for the same protein samples between stained gels and Western blots, it was apparent that the anti-GNA antibody gave a stronger reaction with the 12,000 mol. wt band than with the intact fusion protein; as a consequence, the extent of cleavage of the fusion is overestimated in Western blots.

### Toxicity of recombinant proteins to tomato moth larvae after injection into the haemocoel

The toxicity of recombinant ButaIT was demonstrated by injection into fifth stadium *L. oleracea *larvae (approx. 30 – 60 mg). Injection of the partially purified recombinant protein at doses of the order of 1–10 μg caused a decline in larval suvival over a period of 7 days, to less than 30%. Control survival (buffer-injected, or injected with other partially purified recombinant proteins) was >70% over 7 days. The effect was dose-dependent, and at high doses of ButaIT 100% mortality was observed after 3 days, during which time control survival was >90%. These results could not be compared with previously published data for toxicity of the protein purified from scorpion venom, as the recombinant protein could not be quantified, and attempts to purify it further were not successful. However, the data show that expression as a recombinant protein in *P. pastoris *did not abolish the toxicity of ButaIT.

**Figure 3:**
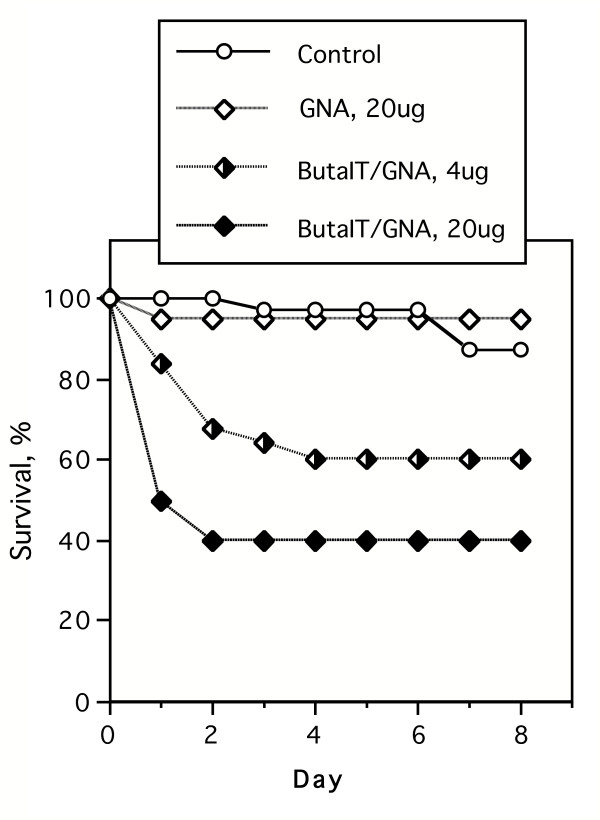
**Survival of *L. oleracea *larvae following injection of purified recombinant proteins**. Survival was recorded for fifth stadium *L. oleracea *larvae following injections of recombinant GNA or ButaIT/GNA. Control insects were injected with phosphate buffered saline (PBS; n = 40). GNA was injected at a dose of 20 μg which equates to approx. 500 μg GNA/g insect (n = 20); ButaIT/GNA was injected at two different concentrations; 4 μg which equates to approx. 100 μg fusion protein or 30 μg toxin/g insect (n = 25), and 20 μg which equates to approx. 450 μg of fusion protein or 135 μg toxin/g insect (n = 10).

The ButaIT/GNA fusion protein was also toxic when injected into 5^th ^stadium *L. oleracea *larvae. The major effect on mortality was seen during the first two days after injection, after which survival (particularly at higher doses) stabilised. The effect was dose dependent; injection of 20 μg of fusion protein per larva (approx. 500 μg per g insect; n = 10) reduced survival to 40% after 6 days, whereas injection of 4 μg of fusion per larva (approx. 100 μg per g insect; n = 25) reduced survival to 60%. Control survival was 97% over this interval (n = 40). Injection of GNA at 20 μg per larva (approx. 500 μg per g insect; n = 20) had no effect on survival, which was 95% over 6 days (fig. 3); thus the toxic effect shown by ButaIT/GNA on injection must be due to the toxin fused to GNA. Surviving insects that had been injected with ButaIT/GNA also showed a reduction in weight gain compared to controls (PBS-injected). The effect was variable, but in a representative assay injection of approx. 100 μg per g insect of the fusion protein reduced weight gain over 6 days by approx. 40% (starting weight 38 ± 2 mg, final weight for controls 205 ± 17 mg, for ButaIT/GNA injected insects 134 ± 19 mg, n = 20 for each treatment, significantly different at p < 0.01). Injection of GNA at similar dose had no effect on weight gain.

### Toxicity of recombinant proteins to tomato moth larvae after oral delivery via artificial diet

**Figure 4:**
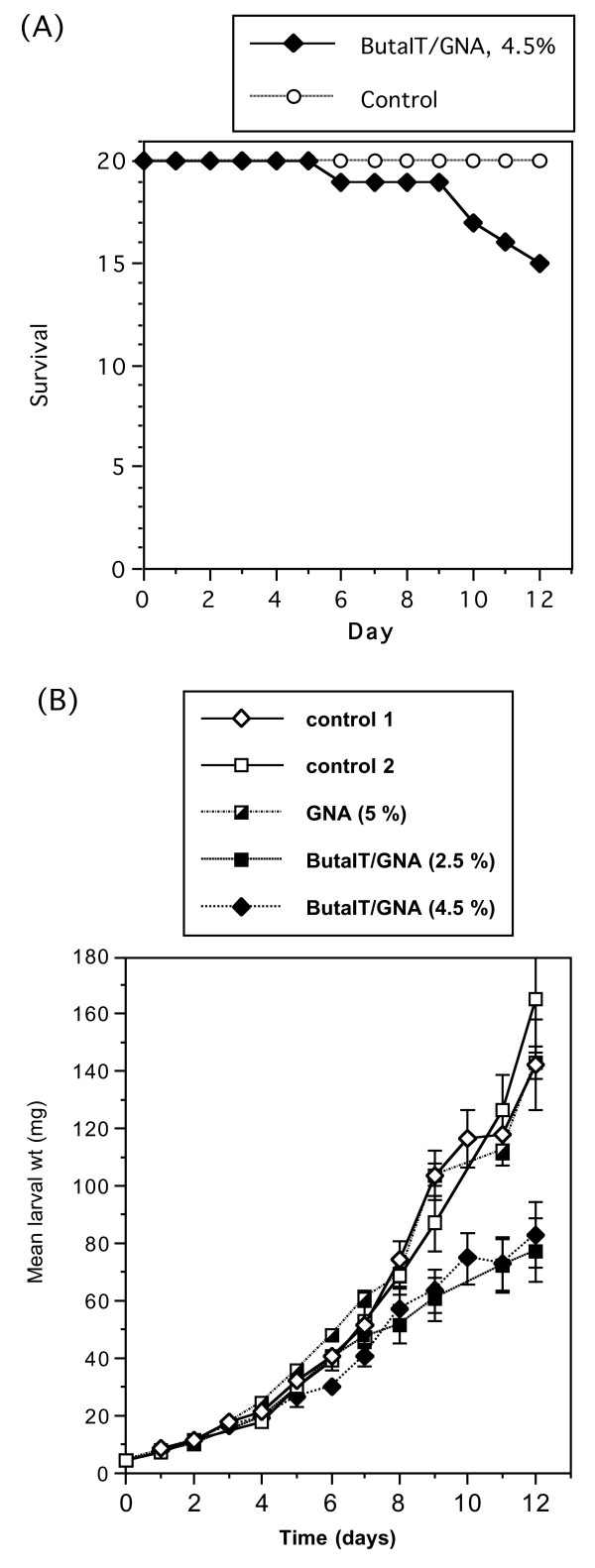
**Survival and weight gain for L. oleracea larvae fed diets containing purified recombinant proteins**. (A). Survival of third stadium *L. oleracea *larvae fed for 12 days on control diet or diets containing recombinant ButaIT/GNA at 4.5 % dietary protein. (B) Mean weight of third stadium *L. oleracea *larvae fed for 12 days on control diet or diets containing recombinant ButaIT/GNA at 4.5 % and 2.5 % dietary protein, or recombinant GNA at 5 % dietary protein. Data has been combined for 2 assays carried out under the same conditions, and thus data for two control treatments is provided. n = 20 per treatment (control total n = 40). Points and bars denote mean ± SE.

Third stadium tomato moth larvae were exposed to diet containing recombinant ButaIT/GNA at 2.5% and 4.5% of dietary protein. Delivery of the toxin via this route had little or no acute toxicity to larvae, but long-term effects on larval survival and development were observed. As shown in Fig 4A, survival of larvae fed on diet containing ButaIT/GNA at 4.5% of total protein declined significantly after day 8 of the assay, so that only 75% of larvae remained alive after 12 days of feeding, compared to 100% of larvae fed on control diet. Exposure to the lower dose of ButaIT/GNA did not have a significant effect on survival over a similar time period. Similarly, exposure to GNA in the diet at 5% of total protein had no effect on survival over 12 days.

Surviving larvae fed on the ButaIT/GNA-containing diets showed a significant reduction in mean larval weight compared to the control diet treatment for all time points after day 4 of the bioassay (ANOVA; p < 0.05), so that by day 12 of the bioassay the mean weight of surviving ButaIT/GNA-fed larvae (both doses) was approx. 40 % less than that recorded for control insects (Fig. 4B). The consumption of diet correlated with larval growth, so that overall, ButaIT/GNA at 4.5 % of total protein caused a reduction of approx. 35 % of total diet consumed, compared to control treatment, and ButaIT/GNA at 2.5 % of total protein caused a reduction of approx. 30% in diet consumption. Feeding GNA at 5% of total protein in the diet had only marginal, insignificant effects on growth and consumption (Fig. 4B).

#### Presence of ButaIT/GNA in tomato moth larval tissues after oral administration

**Figure 5:**
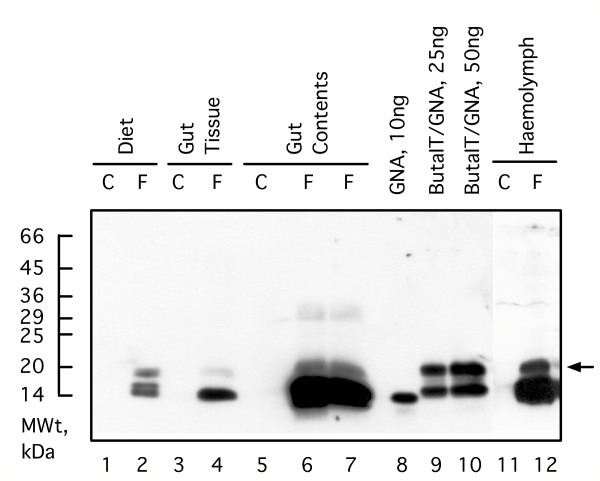
**Presence of ButaIT/GNA fusion protein in gut and haemolymph of *L. oleracea *larvae following feeding**. Samples were taken from third stadium *L. oleracea *larvae fed for 12 days on control diet (C), or diet containing ButaIT/GNA (F; 4.5 mg/5 g diet). Proteins were resolved by 15 % acrylamide SDS-PAGE gel, electroblotted onto nitrocellulose, and probed with anti-GNA antibodies. Loading is as follows: lanes 1, 3, 5 and 11 are control diet, gut tissue, gut contents and haemolymph respectively; lanes 2, 4, 6, 7 and 12 are ButaIT/GNA diet, gut, gut contents and haemolymph; lane 8 is a GNA standard (10 ng); lanes 9 & 10 are ButaIT/GNA standards of 25 and 50 ng, respectively. Loading, as estimated by Bradford assay, was 40 μg total protein for all gut and haemolymph samples.

Gut tissue and haemolymph samples were extracted from larvae exposed to ButaIT/GNA at 2.5 % of dietary protein for 24 h and 8 days, and from larvae exposed ButaIT/GNA at 4.5 % of dietary protein for 12 days. Typically samples were pooled from 3 insects to give 2 – 4 replicates in each instance. Western blot analysis, using anti-GNA antibodies as a probe, was used to verify binding of the fusion protein to the guts of orally exposed larvae, and uptake of ButaIT/GNA into the haemolymph. Fig. 5 represents a summary of the results obtained. In all cases immunoreactivity of two major bands with anti-GNA antibodies was observed in samples from insects fed ButaIT/GNA, but not control insects. These bands, present in both gut and haemolymph samples, correspond in molecular weight to the sizes of ButaIT/GNA and GNA. The susceptibility of fusion proteins based on GNA to proteolytic cleavage in the region of the linker peptide between GNA and the fused peptide/polypeptide has been observed previously for other recombinant fusion proteins [11,12], and has been noted above for ButaIT/GNA. When compared to standards, the ButaIT/GNA fusion protein detected in both gut and blood samples from ButaIT/GNA-fed larvae showed a reduction in the intensity of the band corresponding to intact fusion protein, and a concurrent increase in the band corresponding to GNA. The results implied that limited proteolysis of the ButaIT/GNA fusion occurs during ingestion, passage through the gut, and transport to the haemolymph. Analysis of gut contents from ButaIT/GNA-fed insects by Western blotting suggested that most of the proteolytic cleavage of the fusion protein occurred during the digestive processes occurring in the gut lumen (fig. 5). Analysis of proteins extracted from artificial diet showed that the fusion protein was not proteolytically cleaved as a result of incorporation into the diet, and that it remained intact over a period of 2–3 days (Fig. 5).

#### Effects of ButaIT/GNA on non-lepidopteran insects

**Figure 6:**
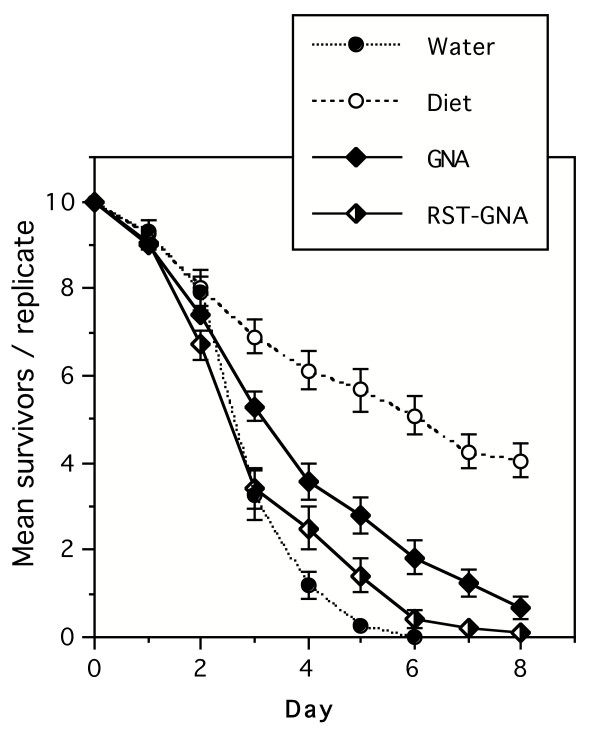
**Effects of purified recombinant proteins on survival of rice brown planthopper**. Third instar juveniles of *N. lugens *were fed on water only (mortality control), liquid artificial diet (negative control) and diet containing recombinant proteins at 0.5 mg/ml (= 0.05% w/v). Graph shows mean survival per replicate of 10 insects (± SE, n = 10 for water, diet, ButaIT/GNA, n = 20 for GNA).

The toxicity of ButaIT/GNA towards non-lepidopteran insects was tested using the homopteran species *Nilaparvata lugens *(rice brown planthopper). The fusion protein was fed to insects by incorporation into liquid diet at a concentration of 0.05% (0.5 mg/ml diet), and its effects on survival were compared to GNA at a similar concentration. Results are shown in fig. 6. Control survival over the 8-day bioassay was approx. 40%, and at day 6, when all insects on the "water only" control had died, was approx. 50%. Corrected mortality values, based on day 6 of the assay, were 64 ± 11% for GNA and 92 ± 5% for ButaIT/GNA; these are significantly different at p < 0.01 (survival analysis). At all times after day 1 the effect of ButaIT on survival was greater than that of GNA in these bioassays. Western blotting analysis of samples of diet after *N. lugens *feeding showed that ButaIT/GNA was stable in the diet over a 2-day interval, with only a small amount of proteolysis of the fusion observed (data not presented).

## Discussion

ButaIT belongs to the sequence family of "short" scorpion toxins, which have a common pattern of cysteine residues and are thought to share a common core structure, containing one α-helix and three β-strands [8]. On the basis that there is more sequence variability at the C-terminus of these proteins than at the N-terminus, where a completely conserved cysteine residue is always amino acid residue 2, the ButaIT/GNA fusion protein was designed with the scorpion toxin N-terminal to the lectin, with the expectation that this orientation would be more likely to result in the fusion protein retaining toxicity. Previous fusion proteins based on GNA have shown that the lectin functionality is retained in both N- and C-terminal fusions [10-12]. Expression of the toxin itself as a recombinant protein containing a C-terminal fusion "tag" sequence would confirm that C-terminal extensions to ButaIT did not abolish toxicity. The availability of a complete cDNA sequence encoding a precursor of ButaIT made assembling a synthetic coding sequence for the mature protein a straightforward exercise.

The high level of cysteine in ButaIT, which is assumed to contain 4 cys-cys cross-links in a 37-residue protein, required that an expression system capable of correctly forming cys-cys cross-links was used to express the recombinant protein. The use of *Pichia pastoris *as an expression host has proved successful in our hands for expressing other cysteine-rich cross-linked proteins, such as the spider venom toxin SflI [11], in a soluble, functional form, and is also able to produce functional, soluble plant lectins [13]. *P. pastoris *was thus used as expression host for both ButaIT and the ButaIT/GNA fusion, with both recombinant proteins engineered for secretion into culture supernatant. Comparison of the toxicity of recombinant ButaIT with the material purified from scorpion venom by HPLC [8] was not possible, since only partially purified recombinant toxin was available. However, the fusion protein was fully purified and the delivered dose could be properly quantified. The ButaIT purified from scorpion venom caused induction of progressive, irreversible paralysis in *Heliothis virescens *larvae 30 min – 24 h after injection at a dose of 1 μg/100 mg insect; in comparison, injection of ButaIT/GNA at 3 – 13 μg/100 mg insect in *L. oleracea *larvae caused mortalities of 40–60%. These data suggest that the toxicity of ButaIT in the fusion protein may be compromised to some extent, but is not abolished, and the fusion protein is still an effective toxin.

Previous studies using recombinant fusion proteins combining snowdrop lectin (GNA) linked either to the insect neuropeptide (Manse-AS) or to an insect spider venom neurotoxin (SFI1) have demonstrated that GNA can be utilised as a transporter to deliver linked peptides to the haemolymph of lepidopteran *L. oleracea *larvae [10,11]. The results presented in this paper provide further verification of the use of GNA for the oral delivery of haemolymph active toxins to the blood of insects. Evidence for binding of the fusion protein to the gut and subsequent delivery to the blood was provided by western analysis of samples taken from fusion-fed larvae. GNA-immunoreactive protein with a molecular weight consistent with the recombinant ButaIT/GNA standard was seen to be present in both gut and haemolymph samples from fusion-fed *L. oleracea *larvae. A degree of proteolytic degradation of the fusion was denoted by the increased intensity of an immunoreactive band of the same size as GNA, concurrent with the decreased intensity of the band corresponding to ButaIT/GNA. The degradation of ingested proteins is part of the digestive process. Whilst GNA itself is resistant to gut proteolysis, proteolytic cleavage of linker regions, or polypeptides fused to GNA is to be expected and has been observed previously [11,12]. Nevertheless, sufficient biologically active ButaIT was delivered to the blood to produce detrimental effects upon larvae exposed to diet containing the fusion protein.

Previous studies with SflI/GNA, a fusion between a spider neurotoxin (SflI) from *S. florentina *and GNA, also demonstrated *in vivo *biological activity by injection of *L. oleracea *larvae. SflI/GNA was seen to cause 40 % mortality 72 hours after injection of a dose of 18 – 25 μg/g insect [11]. This level of toxicity is slightly higher than the effects of ButaIT/GNA on injection. SflI/GNA also showed higher toxicity than ButaIT/GNA when fed to larvae in diet, with 20% mortality over a 4-day period and an almost complete inhibition of growth when fed at levels comparable to those for ButaIT/GNA. When SflI/GNA was fed at lower levels, approx. 1% of total protein, it had no effect on survival, but caused a decrease in weight gain similar to that observed for ButaIT/GNA at approximately 4.5% of total protein. These results are most easily explained by a difference in sensitivity of *L. oleracea *larvae to the two toxins, SflI and ButaIT, with SflI being more potent. In both cases the analysis of haemolymph after feeding fusion protein showed that intact fusion protein was present as a result of GNA-mediated transport across the gut epithelium, and thus oral toxicity would depend on the relative activity of the toxin parts of the two fusion proteins. GNA itself has no acute toxic effects in short-term feeding assays on well-developed larvae.

The bioassays in which ButaIT/GNA was fed to *N. lugens *show unambiguously that the fusion protein is more toxic to these insects than GNA alone. The recombinant protein used for these assays shows a similar level of toxicity to previous assays using GNA purified from *Galanthus nivalis *bulbs [14]. The characterisation of ButaIT previously reported [8] suggested that this toxin is lepidopteran-specific, on the basis of non-toxicity to blowfly larvae. The present results suggest that the specificity of ButaIT may be broader, but could also be interpreted as an effect resulting from the fusion of the toxin to GNA. Injection assays carried out with a range of insects have shown that the ButaIT/GNA fusion protein is toxic to other non-lepidopteran insects (data not presented), and further work will be required to define the range of pest species for which this fusion protein may be an effective insecticide.

## Conclusion

The ability of snowdrop lectin (GNA) to act as a carrier protein to direct the transport of attached molecules into the insect haemolymph has been demonstrated by the production of a recombinant protein containing the lepidopteran-specific toxin from the South Indian red scorpion (*Mesobuthus tamulus*) (ButaIT) fused N-terminally to snowdrop lectin. The fusion protein (ButaIT/GNA) was purified to more than 90 % homogeneity by hydrophobic interaction chromatography and gel filtration chromatography. Biological activity was confirmed by mortality of larvae of tomato moth (*Lacanobia oleracea*) injected with ButaIT/GNA, as compared to controls injected with buffer or GNA (negative controls) or recombinant ButaIT (positive control). The fusion protein showed insecticidal activity when delivered orally to *L. oleracea *larvae; dietary ButaIT/GNA caused a reduction in survival (at higher dose) and a significant reduction in larval growth and consumption, as compared to controls. Intact ButaIT/GNA was present in the haemolymph of insects fed on diet containing the fusion protein, showing that transport from the gut had occurred. The ButaIT/GNA fusion protein was also significantly more toxic than GNA alone when fed to the homopteran plant pest *Nilaparvata lugens *(rice brown planthopper), suggesting that the ButaIT toxin may have a broader insecticidal specificity than initially thought. Fusion proteins of this type have potential applications in agriculture, both as insecticide sprays that can be applied exogenously to crops, and as gene products that can be synthesised endogenously by transgenic plants.

## Methods

### Insects

*Lacanobia oleracea *were reared continuously on artificial diet (Bown et al., 1997) at 25°C under a 16 h:8 h light:dark regime. *Nilaparvata lugens *were kept on 20 day old rice plants (*Oryza sativa *variety 'TN1'), at 28°C, 90% RH, under a 16 h:8 h light:dark regime.

### Materials and recombinant DNA techniques

A cDNA containing the GNA coding sequence has been described previously [15]. Sub-cloning was carried out using the TOPO cloning kit (pCR2.1 TOPO vector) purchased from Invitrogen [16]. *P. pastoris *X33 strain and SMD1168H (Protease A deficient) strain, the expression vector pGAPZαB, and Easycomp *Pichia *transformation kit were also from Invitrogen. Oligonucleotide primers were synthesised by Sigma-Genosys Ltd. [17]. Restriction endonucleases, T4 polynucleotide kinase, T4 DNA ligase, and *Pfu *DNA polymerase, were supplied by Promega [18]. Plasmid DNA was prepared using Promega Wizard miniprep kits. GNA was obtained from Vector Laboratories Inc. or was produced as a recombinant protein in yeast [13]. Anti-GNA antibodies, raised in rabbits, were prepared by Genosys Biotechnologies, Cambridge, UK, and anti-(His)_6 _(C-terminal) antibodies were from Invitrogen.

General molecular biology protocols were as described by Sambrook and Russell [19] except where otherwise noted. All DNA sequencing was carried out using dideoxynucleotide chain termination protocols on Applied Biosystems automated DNA sequencers by the DNA Sequencing Service, School of Biological and Biomedical Sciences, University of Durham, UK. Sequences were checked and assembled using Sequencher software [20] running on Mac OS computers.

### Assembly of expression constructs for recombinant proteins

The ButaIT amino acid sequence (Genbank [AF481881]) was used as the basis for the assembly of a synthetic ButaIT gene. Each strand (i.e. coding and complementary strands) of the sequence encoding the mature ButaIT chain (114 nucleotides) was subdivided into 5 fragments, in such a way that each fragment overlapped neighbouring fragments on the complementary strand by 10–15 bases. Ten oligonucleotide primers based on these fragments were synthesised, and used in an assembly reaction of the full mature ButaIT coding sequence.

The oligonucleotides used were as follows:

Coding strand:

5'-TCGCCTGCAGCAAGGT-3'

5'-GTGGTCCTTGCTTTACAACTGATCCTCAAA-3'

5'-CACAAGCCAAGTGTAGTGAGTGTTGTGGGC-3'

5'-GAAAGGGTGGAGTATGCAAGGGCCCACAAT-3'

5'-GTATCTGTGGTATACAATACGTCGACCGGCAA-3'

Complementary strand:

5'-TTGCCGGTCGACGTATT-3'

5'-GTATACCACAGATACATTGTGGGCCCTTGC-3'

5'-ATACTCCACCCTTTCGCCCACAACACTCAC-3'

5'-TACACTTGGCTTGTGTTTGAGGATCAGTTG-3'

5'-TAAAGCAAGGACCACACCTTGCTGCAGGCGA-3"

The primers at the 5' and 3' ends of the coding sequence contained *Pst *I, and *Sal *I restriction sites (underlined), respectively, which were used to allow subsequent cloning of the ButaIT gene into the expression vector pGAPZαB. All primers were individually 5'-phosphorylated using enzyme T4 polynucleotide kinase. An equimolar solution of phosphorylated primers in standard T4-DNA ligase buffer (without adenosine triphosphate; ATP or dithiothreitol; DTT), was prepared in a final volume of 20 μl. The mix was boiled for 10 min, to denature secondary structures, and slowly cooled to room temperature to allow primers to anneal. After addition of ATP, DTT and DNA ligase, a ligation reaction was carried out for 24 h at 16°C. A PCR reaction was then performed, using sense and antisense primers at the 5' and 3' ends of the ButaIT gene, to obtain sufficient DNA for cloning into the intermediate vector PCR 2.1. The resulting clones were verified by sequencing. Subsequently, the ButaIT coding sequence was excised as a *Pst *I/*Sal *I fragment from PCR2.1, and cloned into the expression vector pGAPZαB to create ButaIT- pGAPZαB.

To create a construct encoding the ButaIT/GNA fusion protein, the sequence encoding the mature GNA peptide (105 residues), derived from LECGNA2 cDNA [15] (Genbank [A18023]), was excised from a previously generated construct (SFI1/GNA-pGAPZαA, [11]) by restriction digestion (5' *Not *I/3' *Xba *I) and ligated into ButaIT-pGAPZαB, which had been digested with the same enzymes. The expressed protein derived from this construct is described in fig. 1.

The construct for expressing recombinant GNA as a control also contained amino acids 1–105 of the mature GNA polypeptide (previously shown to produce a fully active lectin) inserted as an EcoR I – Xba I fragment in the vector pGAPZαA; the expressed protein, after post-translational cleavage of the vector-encoded α-factor prepro-sequence was predicted to contain two extra residues at the N-terminus (EF...) and one at the C-terminus (...D); these extra residues have been shown not to affect activity.

### Expression and purification of ButaIT and ButaIT/GNA

Constructs for expressing recombinant proteins were transformed into *P. pastoris *(SMD1168H strain; ButaIT and ButaIT/GNA or X33 strain; GNA) according to the protocols supplied by Invitrogen [16]. Transformants were selected by plating on media containing zeocin (100 μg/ml). Selected colonies were picked off and confirmed as positive transformants by colony PCR using gene-specific primers. Clones expressing recombinant proteins were identified by immuno-dot blot or Western analysis of supernatants from small-scale cultures, using an anti-(His)_6 _antibody (Invitrogen) for ButaIT, and anti-GNA antibodies for ButaIT/GNA.

For protein production, *P. pastoris *cells containing the ButaIT, ButaIT/GNA or GNA constructs were grown in shake flasks (30°C with shaking), or a BioFlo 110 laboratory fermenter [21] as previously described [11], except that the pH of the growing culture was maintained at 4.0. Recombinant protein samples were purified using hydrophobic interaction chromatography on a phenyl-Sepharose (Amersham-Pharmacia) column, as previously described [11]. Recombinant ButaIT, ButaIT/GNA and GNA eluted at low salt concentration, or in water, and were analysed for purity by SDS-PAGE. Lyophilised ButaIT/GNA and GNA were further purified using gel filtration on a Sephacryl S-200 column (1.6 cm diameter, 90 cm length, 0.3 ml/min), equilibrated in PBS buffer. Fractions containing purified ButaIT/GNA or GNA were pooled, analysed for purity and concentration (absorbance 280 nm; SDS-PAGE), prior to use in injection and diet bioassays. Purified proteins were de-salted by dialysis and freeze-dried, or de-salted and concentrated using Microsep TM centrifugal concentrators (VivaScience AG, Hannover, Germany).

Proteins were analysed routinely by SDS-polyacrylamide gel electrophoresis (SDS-PAGE). Samples were prepared by adding 4× SDS sample buffer (containing 10% 2-mercaptoethanol) and boiled for 5–10 min prior to loading. For separation of low molecular weight polypeptides, SDS-PAGE was carried out using a Tris-Tricine buffer system according to the protocol of Schagger and von Jagow [22]. Gels were either stained with Coomassie Blue, or transferred to nitrocellulose using a Biorad Trans-blot SD semi-dry transfer cell, according to the manufacturers recommendations.

Concentrations of purified recombinant proteins were estimated by comparison with known amounts of standard GNA after SDS-PAGE, or Western blotting using anti-GNA antibodies (1:3300 dilution) as previously described in detail [23]. Haemagglutination assays were carried out as described previously [13].

### Insect Bioassays

Partially purified ButaIT and purified ButaIT/GNA and GNA were tested for biological activity by injecting 5–10 μl of aqueous samples (freeze-dried protein re-suspended in water or PBS) into fifth stadium *L. oleracea *larvae of approx. 50–70 mg in weight. For each concentration tested 10–15 larvae were injected and toxic effects monitored over the next 7 days. PBS or water were injected as negative controls; no difference was observed between these two treatments, and survival over 2–3 days after the injection was routinely >90%.

A potato leaf-based artificial diet [24] was used in assays of the toxicity of recombinant ButaIT/GNA on oral delivery to lepidopteran larvae. For each treatment 20 newly moulted third stadium *L. oleracea *larvae were maintained in clear plastic pots containing moist filter paper to prevent diet desiccation. Survival was monitored daily. Total larval weights (± 0.1 mg) per treatment were recorded daily for the first 4 days of the assay and subsequently, individual larval wet weights (± 0.1 mg) were recorded daily, and diet consumption (per replicate) was estimated on a wet weight basis. The amount of recombinant ButaIT/GNA and GNA added to diets was estimated as described in section 2.4; control diets contained no added protein.

Toxicity of proteins when fed to rice brown planthopper was assayed using a liquid diet. An artificial diet (MMD-1), suitable for the short-term maintenance of *N. lugens*, was prepared according to Mitsuhashi [25]. Assays were set up by placing five second instar *N. lugens *nymphs in each feeding chamber, consisting of the base of a 35 mm petri dish lined with moist filter paper. Two layers of Parafilm M^® ^were stretched over the top, with 100 μl of artificial diet sandwiched between the two layers [26]. Feeding chambers were kept at 28°C, 90 % RH, 16 h:8 h light:dark regime, and the diet sachets were replaced every 2 days to avoid contamination. A total of 10 replicates (50 insects) were used per treatment, and survival was recorded daily over 8 days. The amounts of recombinant ButaIT/GNA and GNA added to diets was estimated as described in section 2.4; diet containing no added protein was used as negative control, and feeding chambers in which no diet was available, but insects were kept moist ("water only") were used as a positive control.

### Analysis of haemolymph and gut tissue in L. oleracea larvae exposed to recombinant proteins

Haemolymph samples were extracted from *L. oleracea *larvae injected with recombinant proteins, or exposed to artificial diet containing ButaIT/GNA or GNA, as previously described [10]. Protein concentrations were estimated by a microtitre-based Bradford assay (Biorad) using BSA as the standard protein. Aliquots of haemolymph were analysed for the presence of GNA immunoreactivity by Western blotting as described previously. Crude gut extracts were also prepared from larvae exposed to ButaIT/GNA diets to verify binding of the fusion protein to the gut epithelium. Whole guts, dissected over ice, were flushed with PBS to remove contents. Following homogenisation, samples were centrifuged (12 000 × rpm, 4°C for 20 mins) and the supernatant assayed for protein concentration prior to analysis by Western blotting. Diet samples were also prepared (as described for gut samples) for analysis by Western blotting to confirm that fusion protein incorporated into artificial diet remained intact in 2–3 day old diet.

### Statistical analyses

All data analysis was carried out using the Statview (v. 5.0; SAS Inc., Carey, NC, USA) software packages on Apple Macintosh computers. Unpaired t-tests, ANOVA analysis (Bonferroni-Dunn), Mann-Whitney non-parametric tests and survival analyses were carried out to determine any significant differences between treatments in the parameters measured.

## Authors' contributions

NPT carried out the molecular biology, preliminary work on protein production and insect bioassay, and insect bioassays using rice brown planthopper; EF carried out bioassays using tomato moth larvae, assisted in protein production, and drafted the manuscript; JAG conceived of the study, designed the cloning strategy for ButIT and the expression constructs, carried out protein purification, and analysed data.
